# Interrupting transmission of soil-transmitted helminths: a study protocol for cluster randomised trials evaluating alternative treatment strategies and delivery systems in Kenya

**DOI:** 10.1136/bmjopen-2015-008950

**Published:** 2015-10-19

**Authors:** Simon J Brooker, Charles S Mwandawiro, Katherine E Halliday, Sammy M Njenga, Carlos Mcharo, Paul M Gichuki, Beatrice Wasunna, Jimmy H Kihara, Doris Njomo, Dorcas Alusala, Athuman Chiguzo, Hugo C Turner, Caroline Teti, Claire Gwayi-Chore, Birgit Nikolay, James E Truscott, T Déirdre Hollingsworth, Dina Balabanova, Ulla K Griffiths, Matthew C Freeman, Elizabeth Allen, Rachel L Pullan, Roy M Anderson

**Affiliations:** 1 Faculty of Infectious and Tropical Diseases, London School of Hygiene & Tropical Medicine, London, UK; 2 Eastern and Southern Africa Centre of International Parasite Control, Kenya Medical Research Institute, Nairobi, Kenya; 3 Evidence Action, Nairobi, Kenya; 4 Neglected Tropical Diseases Unit, Division of Communicable Disease Prevention and Control, Ministry of Health, Nairobi, Kenya; 5 Office of the Executive Committee, Medical Services and Public Health, Kwale County Government, Kwale, Kenya; 6 Faculty of Medicine, Department of Infectious Disease Epidemiology, London Centre for Neglected Tropical Disease Research, School of Public Health, St Mary's Campus, Imperial College London, London, UK; 7 Warwick Mathematics Institute, University of Warwick, Coventry, UK; 8 School of Life Sciences, University of Warwick, Coventry, UK; 9 Faculty of Public Health and Policy, London School of Hygiene & Tropical Medicine, London, UK; 10 Department of Environmental Health, Rollins School of Public Health, Emory University, Atlanta, Georgia, USA; 11 Faculty of Epidemiology and Population Health, London School of Hygiene & Tropical Medicine, London, UK

**Keywords:** EPIDEMIOLOGY, PARASITOLOGY, PUBLIC HEALTH

## Abstract

**Introduction:**

In recent years, an unprecedented emphasis has been given to the control of neglected tropical diseases, including soil-transmitted helminths (STHs). The mainstay of STH control is school-based deworming (SBD), but mathematical modelling has shown that in all but very low transmission settings, SBD is unlikely to interrupt transmission, and that new treatment strategies are required. This study seeks to answer the question: is it possible to interrupt the transmission of STH, and, if so, what is the most cost-effective treatment strategy and delivery system to achieve this goal?

**Methods and analysis:**

Two cluster randomised trials are being implemented in contrasting settings in Kenya. The interventions are annual mass anthelmintic treatment delivered to preschool- and school-aged children, as part of a national SBD programme, or to entire communities, delivered by community health workers. Allocation to study group is by cluster, using predefined units used in public health provision—termed community units (CUs). CUs are randomised to one of three groups: receiving either (1) annual SBD; (2) annual community-based deworming (CBD); or (3) biannual CBD. The primary outcome measure is the prevalence of hookworm infection, assessed by four cross-sectional surveys. Secondary outcomes are prevalence of *Ascaris lumbricoides* and *Trichuris trichiura*, intensity of species infections and treatment coverage. Costs and cost-effectiveness will be evaluated. Among a random subsample of participants, worm burden and proportion of unfertilised eggs will be assessed longitudinally. A nested process evaluation, using semistructured interviews, focus group discussions and a stakeholder analysis, will investigate the community acceptability, feasibility and scale-up of each delivery system.

**Ethics and dissemination:**

Study protocols have been reviewed and approved by the ethics committees of the Kenya Medical Research Institute and National Ethics Review Committee, and London School of Hygiene and Tropical Medicine. The study has a dedicated web site.

**Trial registration number:**

NCT02397772.

**Strengths and limitations of this study**

- The study has a strong design incorporating random allocation, blinding of assessors to the primary outcome, and builds on and will, subsequently, refine mathematical modelling.
- The interventions include alternative treatment strategies using two different delivery systems, and are well-established, of long duration (24 months) and nested within an ongoing national control programme.
- The study includes cost-effectiveness analysis and analysis of community acceptability, feasibility and scale-up of each delivery system.
- A limitation of the study is its reliance on existing health structures to implement the intervention.
- The study will contribute to evidence regarding the cost-effectiveness of soil-transmitted helminth control and thereby inform national and global policy.

## Introduction

Neglected tropical diseases (NTDs) are a cluster of tropical diseases that affect more than one billion people worldwide, mainly among poor populations living at the periphery of health systems.^1^ NTDs can cause disability, disfigurement, undernutrition and cognitive impairment, yet many NTDs can be easily controlled by periodic mass treatment using safe and broad spectrum drugs. Global efforts to control NTDs reached a turning point in 2012, when WHO launched its NTD Roadmap,^1^ and partners met in London, and pledged to work together to control and eliminate 10 NTDs by 2020.^2^ As part of this commitment, pharmaceutical companies pledged to donate the drugs required for mass treatment programmes and the challenge now is to support countries in developing sustainable systems to distribute donated medicines.

According to the 2010 Global Burden of Disease study,^2^ the soil-transmitted helminths (STHs) spp *Ascaris lumbricoides*, *Trichuris trichiura* and hookworm, contribute the greatest disease burden among the NTDs, causing an estimated 4.98 million years lived with disability each year.^3^ Fortunately, much of this burden can be readily averted by periodic, population-based chemotherapy (also known as deworming). The WHO identifies three priority groups for deworming: school-age children, preschool-age children and women of childbearing age,^4^ as they typically harbour chronic and intense infections at a time when they are undergoing physical and/or cognitive development. An effective mechanism to reach school-age children is provided by school-based deworming programmes, which have been shown to cost-effectively reduce their STH-related morbidity.^5^ ^6^ In 2013, some 237 million school-age children—equivalent to 39% of the global at-risk school-aged population—benefitted from STH treatment.^7^ However, if the 2020 target of treating 75% of school-age children is to be reached, there needs to be a concerted effort to scale-up deworming. Responding to this need, a new consortium was established in Paris in 2014, to assist countries to develop mechanisms for addressing STH among preschool-age and school-age children.^8^ Partners at the Paris meeting also committed support for evaluating the potential of interrupting the transmission of STHs using new tools and strategies. Recent analyses based on mathematical models of parasite transmission and the impact of treatment have suggested that the transmission of STHs can be interrupted (a breakpoint in transmission is crossed where parasite elimination is achieved) if treatment is expanded to adults and provided more frequently.^9–11^ While such models can provide new insights, there is an obvious need to test the predictions of the impact and cost-effectiveness of alternative treatment strategies through rigorous field studies.

If there is to be a move towards broadening the range of age groups targeted by STH treatment programmes, then it is necessary to identify suitable delivery systems. Possibly the longest running community-based NTD control programme that treats across all age groups is the African Programme for Onchocerciasis Control, which has helped countries create a community-directed treatment strategy by involving community-directed drug distributors and extending and strengthening health systems.^12–14^ A community-based approach is also employed by national lymphatic filariasis (LF) control programmes, whereby community drug distributors or community health workers (CHWs) provide community-wide delivery of albendazole plus ivermectin (or diethylcarbamazine in areas not endemic for onchocerciasis) to entire populations aged 2** **years and above.^15^ Although CHWs are increasingly used to promote healthy behaviours and deliver basic health services, especially in poor and underserved communities,^16^ ^17^ the benefits of using a CHW-based approach for STH control is poorly understood at present.

This paper describes two cluster randomised trials in Kenya that seek to provide new evidence on the impact and cost-effectiveness of alternative treatment strategies and delivery systems in reducing the transmission of STHs. Such evidence will help establish proof-of-concept of the possibility of interrupting STH transmission and would likely be of value to policymakers in STH-endemic countries, and partners and funders supporting STH control.

### Aims and objectives

The overall aim of the trials is to evaluate the impact and cost-effectiveness of school-based versus community-based deworming on measures of STH transmission in Kenya. Specifically, we will test the hypothesis that treatment needs to be provided to a broad range of ages and/or at more frequent intervals than 1 year in order to interrupt the transmission of STH. The study also includes process and economic evaluations to assess the feasibility and implementation of the alternative treatment strategies and delivery systems, which will guide scale-up of the programmes in Kenya and other settings in Africa. The more detailed study objectives are:

1. To quantify the impact of school-based versus community-based mass treatment (treatment strategies and delivery systems) at annual and biannual intervals (treatment strategies) in reducing the transmission of STH spp, hookworm, *A. lumbricoides* and *T. trichiura*.
2. To evaluate the costs and cost-effectiveness of alternative STH treatment strategies and delivery systems in reducing transmission.
3. To assess the extent to which community-based treatment programmes for STH are acceptable to the community, which are feasible, given the health system capacity, and can be easily scaled-up across Kenya and elsewhere.

## Methods and analysis

Reporting of the study protocol has been verified in accordance with the SPIRIT (Standard Protocol Items for Randomised Trials) recommendations.

### Overall study design

Two paired community cluster randomised trials in different settings in Kenya will evaluate the impact and cost-effectiveness of annual school-based deworming, annual community-based deworming and biannual community-based deworming. The trials are designed as cluster randomised, open-label trials with three study groups. The primary outcome is the prevalence of hookworm. This outcome was selected because it is the STH spp that contributes most to morbidity, is responsible for the most DALYs lost due to STH^3^ and is the species most difficult to control using school-based deworming alone.^9^ Allocation to study group is by cluster, using predefined units used in public health provision—termed community units (CUs). The three study groups are:

1. *Control group*: Annual school-based deworming. Preschool and school children (typically aged 2–14 years) will receive a single dose of albendazole (400 mg) from trained school teachers, as part of the ongoing national school-based deworming programme.
2. *Expanded age range group*: Standard school-based deworming supplemented by annual community-based deworming (2–99 years). All household members who are not enrolled in school will receive a single dose of albendazole (400 mg) from trained CHWs—known in Kenya as community health volunteers (CHVs).
3. *Expanded age range and frequency group*: Annual school-based deworming supplemented by community-based deworming (2–99 years), followed by an additional community-based deworming 6 months later. All household members who are not enrolled in school will receive a single dose of albendazole (400 mg) from trained CHVs.

Mathematical models suggest that there is little difference in the impact of annual or biannual school-based deworming, given the expected prevalence in our study sites,^10^ ^18^ and therefore we did not include biannual school-based deworming as a study group. In other settings where *A. lumbricoides* is more common and hookworm is absent, biannual treatment may be desirable due to the relatively higher levels of infection in school-age children compared to adults.

**Figure 1:**
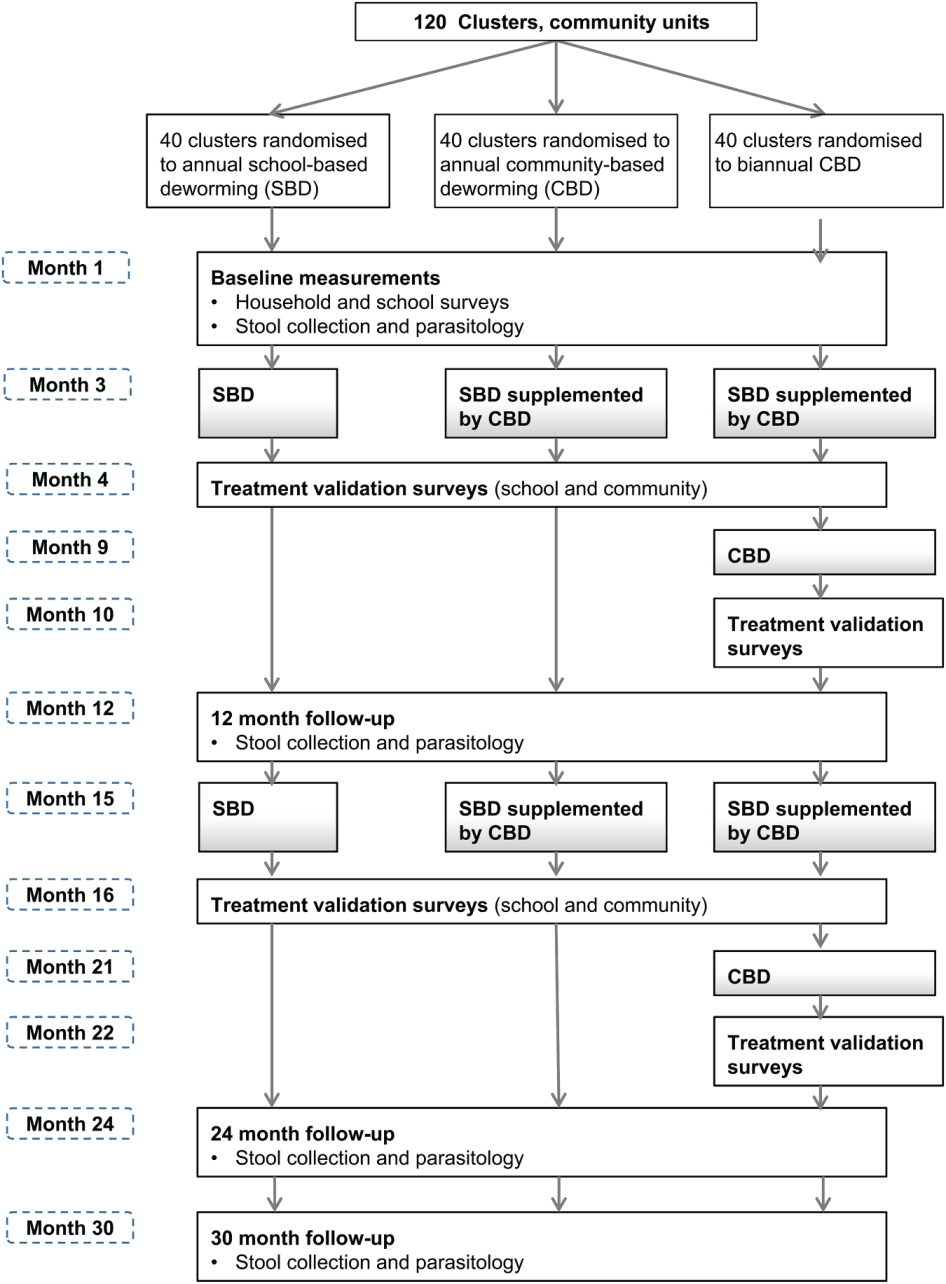
Summary of study design.

The primary outcome, the prevalence of hookworm infection, will be measured through cross-sectional parasitological surveys conducted at baseline and at 12, 24 and 30 months follow-up. The timing of the final follow-up survey takes into account differences in time since treatment of the annual and biannual treatment groups at 24 months. The overall study design is summarised in figure 1. A subsample of individuals from two CUs in each of the study groups will be followed longitudinally for two and half years, in order to better understand the transmission dynamics of STHs and to estimate key parameters for the mathematical models of transmission dynamics and treatment impact. A nested process evaluation, using semi-structured interviews, focus group discussions (FGDs) and a stakeholder analysis, will investigate the community acceptability, feasibility, given the local and regional health system structures and processes, and scale-up of the interventions.

### Interventions

All study groups will receive treatment with albendazole (400 mg), which is highly efficacious against *A. lumbricoides* and hookworm, but has lower efficacy against *T. trichiura.*^19^ What differs between the three study groups is the age range of populations that receive treatment and the frequency at which treatment is provided.

School-based deworming will be implemented in all communities as part of the national school-based deworming programme (NSBDP). Launched jointly in 2009 by the Ministry of Education, Science and Technology (MoEST), and the Ministry of Health (MoH), and funded by the Children's Investment Fund Foundation, the programme's goal is to eliminate STHs and schistosomiasis as a national public health problem. A single 400 mg dose of albendazole is provided annually by trained teachers on designated deworming days. The programme targets all children in at-risk subcounties enrolled in public and private primary schools and early childhood development centres (kindergartens).^20^ Non-enrolled school-age children are also encouraged to come to school for treatment on deworming day. The NSBDP makes use of a cascade model whereby trainings, materials, drugs and funds are channelled from the national level through the county to the school level, involving officials from MoEST and MoH throughout the cascade. The emphasis is on MoEST for delivery and MoH for supervision. Treatment coverage data and remaining drugs are returned along the reverse cascade. No adaptations shall be made to the NSBDP for the study other than modification of the treatment and coverage forms to strengthen the data capture on treatment at the individual level, and to validate treatment coverage.

Community-based deworming will be provided by CHWs, called CHVs in Kenya. CHVs already promote care seeking and compliance to antiretroviral and tuberculosis treatment, and mobilise populations during national health campaigns, as part of the national community health strategy.^21^ This strategy also includes the establishment of CUs, which each serve approximately 1000 households or 5000 people, with a single CHV providing service to approximately 100 households. For every 25 CHVs, there is one Community Health Extension Worker (CHEW) who is a Ministry of Health employee with training in public health or nursing. This CHEW provides supervision and technical support to the CHVs. The community health strategy was revised in 2010, with new guidance on the contents of CHV kits to include basic drugs such as paracetamol, albendazole and tetracycline.^22^ The community-based deworming in the present trials will provide door-to-door treatment, drawing on the previous experience of the National Programme for Elimination of LF, which, to date, has implemented four rounds of mass treatment (2002, 2005, 2008 and 2011), but with variable levels of treatment coverage.^23^

### Setting

**Figure 2:**
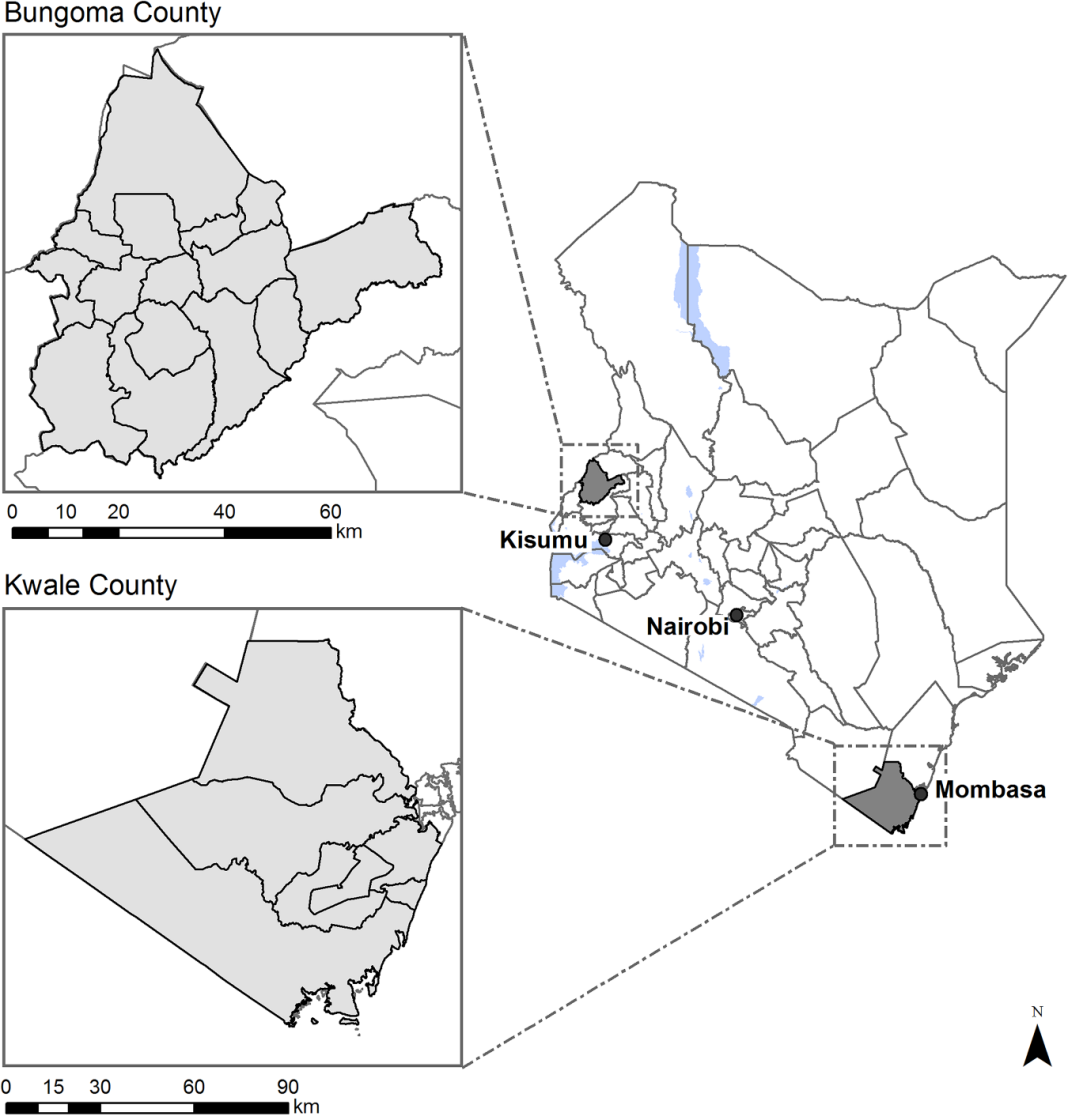
Map showing location of study sites and community units.

The study will be conducted in two settings of Kenya that have contrasting epidemiological and programmatic characteristics: Kwale County on the south Kenyan coast and Bungoma County in western Kenya (figure 2). Key epidemiological and sociodemographic characteristics of the two study areas are summarised in table 1. Historically, STH infections have been highly prevalent in both regions but recent control efforts have reduced levels of infection. *A. lumbricoides* is more common in western Kenya, whereas hookworm predominates on the coast.^20^ *T. trichiura* is present in both settings, but at low levels. In Kwale County, in addition to STHs, there is focal transmission of *Schistosoma haemotobium*, and LF is endemic.^24–26^ In relation to sociodemographic characteristics, Kwale County is among the poorest in Kenya, with low levels of access to water and sanitation, and minimal primary school enrolment, while Bungoma lies close to national averages for these factors.

**Table 1:** Epidemiological and socioeconomic characteristics in the two study areas

	Bungoma	Kwale County	National average	Source
Helminth infections				
STHs combined (%)	49.3	33.6	32.4*	27
Hookworm (%)	44.3	27.7	15.6*	27
* Ascaris lumbricoides* (%)	28.2	0.8	18.0*	27
* Trichuris trichiura* (%)	0.8	8.9	6.6*	27
* Schistosoma haematobium (%)*	non-endemic	17.5	14.8*	27
* Wuchereria bancrofti* (%)	non-endemic	endemic	endemic in 6 of 47 counties	28
Socioeconomic conditions				
Poverty rate (%)†	52.2	72.9	46.6	29
Access to water and sanitation				
Improved drinking water (%)	72.1	51.2	55.1	30
Improved sanitation (%)	71.2	34.4	64.9	30
School system				
Primary school attendance (%)‡	94.6	87.2	85.6	30
Literacy rate (%)	60.5	66.5	66.4	31
Health system				
Full immunisation coverage (%)§	84.4	77.5	83.0	32
Doctors (per 100 000 people)	4	1	7	32
Nurses (per 100 000 people)	37	37	49	32

*Among schools included in the monitoring and evaluation of the national school-based deworming programme.

†Percentage of population living below the Kenya poverty line (Ksh 1562 per person per month in rural areas and Ksh 2913 in urban areas).

‡Percentage of the official primary school-age population that attends primary school.

§Percentage of population that completed 3+ doses of diphtheria, pertussis and tetanus vaccination.

STH, soil-transmitted helminth.

### Randomisation

Allocation to study group will be by cluster, using CUs. In areas where there are no formal CUs in existence, we worked with the public health officers and CHEWs to delineate CUs in order to ensure that every village and government is assigned to a CU. Randomisation was stratified by the prevalence of hookworm (below and above 20% prevalence, as determined in the baseline survey), and subcounty and size (below and above 840 households), in order to reduce the likelihood of chance imbalances. Randomisation took place at public ceremonies. The randomisation sequence generation was undertaken by an independent statistician using computerised random number generation. Sealed envelopes containing CU identification were placed in prestratified ballot boxes, with delegates invited to select envelopes from the boxes and directed to put the selected envelope in a box labelled A, B or C (corresponding to the three study groups trial) according to the pregenerated randomisation sequence for that stratum. In each CU, 225 households are randomly selected and one randomly selected household member is recruited into the cross-sectional surveys.

Owing to the nature of the interventions, participants are not blinded to their group randomisation. However, the identity of the study groups remains hidden until the completion of community sensitisation and randomisation to eliminate participation bias. In addition, the laboratory technicians conducting stool examinations and the statistician responsible for analysis are blinded to the group assignment.

Contamination between clusters may occur when people from one cluster receive treatment implemented in another cluster or have lower exposure to STH infection due to lower transmission in another cluster. Our use of CUs, which comprised groups of villages rather than single villages, helps reduce the possibility of contamination. CUs, including CUs located in urban and periurban areas, which generally are not comprised of distinct groups of villages, were excluded.

### Sensitisation and recruitment

Key stakeholders and policymakers have been involved in the study and its design from conceptualisation. Meetings have been held with the MoH and MoEST in Nairobi, and at each study site, where key stakeholders were sensitised about the study objectives, intervention and evaluation procedures, and requested to provide input. Community meetings were held to describe the purpose of the study, the interventions, the evaluation procedures to be followed, and the risks and benefits of participation. Individuals had the opportunity to ask questions. Consent for the intervention was provided at the community level with the option for individuals to opt-out either from receiving treatment or study assessments.

Consent for the baseline and follow-up cross-sectional surveys is obtained at the individual level. Field staff enumerate all households through coordination with the chiefs and village elders. Subsequently, households selected for inclusion into the study are visited by field staff. In each household visited, written informed consent to conduct the household-level questionnaire is sought from the household head. Following this, a random function programmed in a smartphone selects the individual within the household who will provide a stool sample and answer further individual-level questions. Individual-level informed consent is sought from selected individuals (either for themselves or their children) and written assent sought from children over 13 years of age. Inclusion criteria for the selection of individuals include: (1) resident for at least 12 months, (2) willingness of adult aged 18 years and above or parent/guardian to provide written informed consent, and (3) provision of written assent to participate from children aged between 13 and 17 years. Exclusion criteria include: (1) recent (<12 months) resident or visitor to household at time of household visit, (2) refusal of informed consent and (3) refusal to assent by children aged 13–17 years.

### Outcomes

The primary outcome is the prevalence of hookworm (*Necator americanus* or *Ancylostoma duodenale*) infection among all sampled individuals during 30 months of follow-up. Owing to ethical considerations of treating those found infected during surveys, new populations of individuals will be selected for each cross-sectional survey (baseline, 12, 24 and 30 months). All participants are asked to provide a stool sample, which is examined in duplicate using the Kato-Katz method. Individuals found infected are revisited by the study team and treated with albendazole. In a random subset of individuals, additional confirmatory diagnosis of infection is based on real-time PCR, which also allows the differentiation between hookworm spp.^33^ ^34^

The main secondary outcomes include:

- Prevalence of *A. lumbricoides* and *T. trichiura*, based on expert microscopy and, in a random subsample, on real-time PCR.
- Intensity of infection for each STH spp, based on quantitative egg counts.
- Treatment coverage, measured using both routine data, and data collected during household visits to track treatment coverage and compliance.

### Survey procedures

At each house visited, household heads are interviewed to collect a household census and information on household characteristics and ownership of key assets during household visits. Data on household water, sanitation and hygiene (WASH) conditions and school WASH conditions are collected using structured observations and questionnaires, employing tools piloted and extensively used in Kenya.^35^ ^36^

Teachers and CHVs will be provided with treatment registers and asked to provide a full record of all individuals who have received treatment. To augment these data, population-based coverage surveys using multistage clustering sampling^37^ will be carried out among a random subsample of communities.

In each cross-sectional survey, a randomly individual selected is asked to provide a stool sample, which is transported to a nearby health facility laboratory and examined in duplicate within 1 h of processing using the Kato-Katz method. Duplicate slides are read by independent microscopists. A 10% quality control check is performed by a supervisor. Aliquots of randomly selected stool samples are preserved in ethanol for confirmatory real-time PCR diagnosis.^33^ ^34^ In addition, stool samples will be stored for future molecular analysis, including the detection of potential drug resistance alleles,^35^ ^38^ and genome sequence analysis to investigate the genetic structure of helminth populations.^39^

All members of the study teams have been appropriately trained in the study objectives and procedures. Standard operating procedures have been developed, field-tested and revised, and are used to guide all field activities. Supervisors make regular visits to the field to monitor fieldwork.

### Longitudinal studies

In six CUs (one with a medium (20–49%) and one high (>50%) prevalence of hookworm in each study group), individuals will be followed longitudinally to help quantify the transmission dynamics of the parasites and re-parameterise mathematical models of transmission dynamics. An age-stratified random sample of 200 individuals will be chosen and asked to provide complete stool samples for a period of 5 days immediately following treatment. The collected stool will be transported to an off-site sorting facility, *A. lumbricoides* and hookworm will be manually separated from the stool, and the number and sex or worms recorded for each individual. The proportion of unfertilised eggs will also be determined for *A. lumbricoides*, given the importance of this measure as a determinant of how effective a given treatment programme is in driving transmission to extinction by crossing the transmission threshold where insufficient mating occurs to sustain transmission.^40^ Selected individuals will be revisited at 1, 3, 6, 9 and 12 months post-treatment and asked to provide a stool sample, which will be examined for the presence of STH eggs in duplicate using the Kato-Katz method.

We will also conduct household visits to assess the extent of non-compliance to treatment and factors associated with non-compliance.^37^ Adverse events will be monitored in these cohorts during household visits. Reports of severe adverse events that are classified as at least possibility related to the study drugs will be reported to the ethics committee.

### Sample size calculation

**Figure 3:**
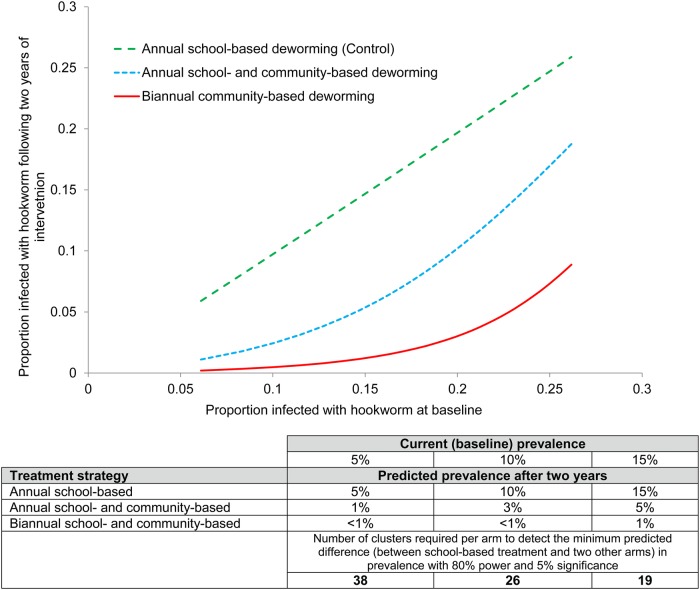
The relationship between baseline prevalence of hookworm infection (proportion of all community members found to be infected) and predicted impact following 2 years of treatment for each proposed treatment strategy. Based on a mathematical model of transmission dynamics, assuming 80% treatment coverage of school-based deworming and 70% of community-based treatment. Biannual school-based treatment did not differ significantly from annual school-based treatment and therefore is not shown. Sensitivity of diagnosis is assumed to be 63%.

Sample size calculations are based on the principles of cluster randomised trials.^41^ Analysis of survey data collected as part of the ongoing monitoring and evaluation of the NSBDP suggests that the prevalence of hookworm, the primary outcome, varies between 5% and 10%, with an intracluster correlation coefficient (ICC) of 0.125.^2^ The assumed difference between the study groups in the prevalence of hookworm after 30 months is based on mathematical modelling of the predicted impact of different treatment strategies,^11^ ^12^ and is presented in figure 3 for different degrees of prevalence of infection at baseline. The figure also presents the number of clusters required to detect the smallest predicted difference between school-based treatment and two other arms with 80% power a 5% level of significance, an ICC of 0.125 and 225 participants per cluster. Based on these calculations and taking into account potential loss to follow-up of CUs (assumed to be 5%), a conservative sample size of 40 clusters per group will be used in both study areas.

### Data management

Data are collected in the field using Samsung GT7552 smartphones running android operating system V.4.2. The questionnaires are programmed using Survey CTO software (http://www.surveycto.com) and data are downloaded daily using secure Wi-Fi in the field office into a web-based database. Backup of the database to a central server is performed daily. Laboratory results are recorded in laboratory books by technicians and double-entered into a customised database and saved on a centralised server.

### Data analysis

Primary analysis will be carried out on groups as randomised (intention-to-treat). Results will be presented as appropriate effects sizes with a measure of precision (95% CIs). Clustering by CU will be included in all analyses. Our main analysis of the primary outcome, the prevalence of hookworm, and other secondary outcomes, will be based on cross-sectional analyses comparing the outcome at 30 months follow-up between study groups. Unadjusted and adjusted results will be presented for all analyses. Covariates in adjusted analyses will be specified a priori and will include subcounty and urban/rural classification, household socioeconomic status, and access to adequate water and sanitation. For continuous outcomes, analyses will adjust for baseline by inclusion of the cluster mean of the outcome in question as a covariate in statistical models. For binary outcomes (notably the primary outcome), no baseline adjustment will be made because of issues relating to the non-collapsibility of ORs.

Demographic and other baseline characteristics of clusters will be compared to check for imbalances between study groups. Tabulation of these measures will be generated using the intention to treat data sets. No significance tests will be performed to investigate for differences between groups at baseline. Where imbalances are suspected, further exploratory adjusted analyses will be carried out that include additional adjustment for these factors. Formal statistical testing will be restricted to comparison between the two community-based treatment groups and the school-based treatment group (control). A small number of secondary outcomes will be prespecified for statistical testing along with the primary outcome. No formal adjustment will be made for multiple testing but the number of outcomes formally tested will be restricted to fewer than 10 and the results interpreted with due caution. This includes multiplicity associated with the two experimental arms. No formal comparisons will be made between the two experimental arms as the study has not been powered for this analysis.

A small number of secondary subgroup analyses will be specified in advance and will be carried out using formal statistical tests for interactions. These will include household poverty, remote households, frequent non-compliers of treatment, non-enrolled children, and households without access to adequate water and sanitation. These analyses will help understand the impact heterogeneity of the interventions.

### Process evaluation

A key study objective is to understand lessons for the scale-up of community-based deworming in Kenya and elsewhere in Africa. Therefore, a qualitative evaluation will seek to identify and describe key assumptions and conditions underlying the implementation, sustainability and scaling-up of the different strategies and delivery systems. The focus of the evaluation will centre on whether CHVs can be utilised for the effective delivery of chemotherapy for control of STH and what factors influence the use of CHVs, including what type of incentives, if any, should be given. Investigation will focus on: (1) community acceptability, which will be assessed during FGDs and in-depth interviews (IDIs); and (2) feasibility, including a situation and stakeholder analysis of the structural, organisational and management factors that enhance or constrain effective implementation.^42^ A series of FGDs will be conducted with community members, teachers, CHVs, CHEWs and local health officials, to better understand the acceptance and implementation of the interventions, using predefined and structured topic guides. FGDs will be stratified by location and socioeconomic status, and specific efforts will be made to reach groups that may be marginalised due to their economic and sociocultural position. IDIs will be carried out with a range of actors and opinion leaders in order to understand the process and constraints of the different delivery systems. The number of IDIs will depend on when saturation is reached, but they will include members of the county, and national health and education teams.

FGDs and interviews will be digitally recorded, with notes additionally taken, transcribed and translated. Transcripts will be imported into NVivo (QSR International, Doncaster, Australia), coded by two independent coders, and analysed using content analysis to identify emerging themes.^43^ Following descriptive analysis, patterns and linkages among views, experiences and behaviours of the participants will be explored. The collected data will provide important contextual information and a basis for evaluating the generalisability of the study findings. The work will benefit from previous qualitative evaluations by the research teams in the study areas.^44–47^

### Cost analysis

Cost data will be collected following an ingredients approach, based on a semistructured questionnaire and by consultation of the programme accounting system. Data collection will be based on a standardised costing framework, capturing fixed and recurrent costs incurred at school and community levels. The questionnaire will include both cash and in-kind contributions, and will be used to estimate financial and economic costs of the alternative treatment strategies (annual vs biannual deworming) and delivery systems (through schools by teachers and through door-to-door delivery by CHVs). Financial costs capture actual expenditures in terms of programme implementation, whereas economic costs include opportunity costs of teachers, CHVs and other stakeholders in delivering deworming. Opportunity costs of the government staff and community members will be calculated using local pay scales. Capital costs will be annuitised over the useful life of equipment, vehicles and other assets using a discount rate of 3%. Costs will be assessed from a societal perspective. Analysis of costs will be linked to volume of treatment in order to determine cost functions. The work will also present an analysis of the full cost of running national programmes in Kenya. Itemised-costing and sensitivity analysis will enable estimation of the costs of scaled-up implementation, and implementation in settings with different epidemiological and programmatic characteristics.

### Mathematical modelling and cost-effectiveness analysis

The questions being addressed in the trial arose from analyses of the predicted impact of mass treatment based on mathematical models of the transmission dynamics and control of STHs.^9–11^ ^48^ The potential for transmission of STHs and other helminths in a defined setting can be quantified by the value of the basic reproductive number (R_0_), which is defined as the average number of offspring produced by one female worm that survives to reproductive maturity. R_0_ can be stratified by age (age-related exposure), and various environmental and behavioural factors.^49^ In endemic communities, mathematical models of helminths that are dioecious have two stable points (no parasites and a stable endemic equilibrium of parasite persistence), separated by an unstable point—the so called ‘breakpoint’ in transmission (a point at which R_0_ falls to just above but close to unity (1) in value), below which continued treatment can quickly drive the population to extinction. For attempts at elimination, the goal of mass treatment is to drive the parasite population below the ‘breakpoint’ by treating sufficient fractions of the target population. Models of STH transmission provide insight into optimal treatment strategies for achieving this breakpoint,^9^ ^10^ and show that annual or biannual community-based deworming can reduce overall prevalence and associated intensity substantially. As an example, reaching the transmission breakpoint can be achieved after 2 years given a baseline prevalence of 10% or less (low transmission setting) if coverage is high. The models also highlight how the age-distribution of worm burden determines the breadth of age groups that should be treated and the importance of considering the species mix.

Data arising from the trials will be used to validate the initial model predictions and to provide better estimates of key epidemiological parameters for use in model predictions. Specifically, the study will produce better estimates of the following parameters: density dependent fecundity, parasite distributions in the various age groupings (estimates of the negative binomial aggregation parameter k), age dependent exposure to infection, drug efficacy and treatment compliance. The models will be fitted with estimates of age-stratified patterns of reinfection and intensity of infection, as well as estimates of treatment coverage using Monte Carlo Markov Chain methods. Such model fitting and parameter estimation will allow examination of whether the observed and predicted impact is consistent. A stochastic model of STH transmission is also under development to enable estimates of variability around deterministic predictions (such as time to crossing the breakpoint) to be made taking account of the many heterogeneities involved in parasite transmission.

The models will be used to explore the impact of the different treatment strategies in a range of settings, with different underlying intensity in levels of transmission and treatment coverage, and the potential impact of alterations (in coverage and frequency) to the treatment strategies over time. The duration of treatment required to cross the ‘breakpoint’ in transmission will also be examined. Particular attention will be given to non-compliers to treatment (both persistent and irregular) in all study groups, and work will examine the impact on overall transmission of poor adherence to treatment.

The cost data, detailed above, will be integrated into the models for cost-effectiveness analysis. The incremental costs per infection, heavy infection, anaemia case and disability adjusted life years averted will be estimated for the two new strategies compared to the current situation with annual school-based deworming. Sensitivity analysis will be undertaken to account for uncertainties in the analysis, with attention given to the impact of non-compliance.

## Ethics and dissemination

### Ethical approval

The study has been approved by the Kenya Medical Research Institute and National Ethics Review Committee (SSC Number 2826) and the London School of Hygiene and Tropical Medicine (LSHTM) Ethics Committee (7177). Sponsorship and insurance is provided by the LSHTM's Clinical Trials Sub-Committee (QA615).

### Informed consent

The study is intentionally embedded within the ongoing NSBDP, which will continue to deliver deworming to all schools in the study areas. At the time of household visits, household members are asked to give their verbal consent for their participation in the community-based deworming. Written informed consent is obtained from adults and parents or guardians of children, before enrolment in the cross-sectional surveys and longitudinal surveys. Written informed consent will also be sought from individuals included in the qualitative evaluations, including FGDs and in-depth interviews. Participants of FGDs and interviews will be provided the options not to be quoted in any reporting of findings. Study information sheets are provided in English, Kiswahili, Mijikenda, Luhya or Bukusu. Translated documents were verified through back-translation into English. Written assent to participate in the cross-sectional and longitudinal studies is obtained from children aged 13 years and above.

Risks and benefits of participating in the study are presented during community meetings and any issues arising discussed. The risk of participating in the trial is very low. The study drug, albendazole, is extremely safe and no severe adverse events are expected.^50^ In the unlikely situation of events occurring, these will be reported to the study site investigator and the principal will inform the ethics committee. Collection of stool samples is a routine procedure and is considered not to be a medical risk; there is the possibility of embarrassment, which will be minimised by appropriate action.

All information will remain confidential. Laboratory specimens, reports, data collection, and process and administrative forms will be identified by a coded unique identifier to maintain participant confidentiality. Access to collected data will initially be limited to fieldworkers at the point of data collection, and to the study statistician and investigators during analysis. As indicated below, data that are considered non-sensitive and do not include identifying participant information will be made publicly available once the main findings have been published, subject to appropriate data sharing agreements.

### Trial oversight

No data safety and monitoring board will be established since the interventions are extremely safe and already delivered to hundreds of millions of individuals each year as part of national deworming programmes. Instead, an independent Data Monitoring Committee (DMC), consisting of a chair and three members, and operating under a remit of a charter, has been established to monitor data for quality and completeness. The DMC will review, in strict confidence, an interim analysis of the 12-month data. The DMC will also review and approve the final data analysis plan.

### Dissemination

The study has a dedicated web page, on the Global Atlas of Helminth Infection (GAHI) website (http://www.thiswormyworld.org/tumikia-project), where study updates and final results will be disseminated. Study findings will also be disseminated through multiple and innovative media, ensuring that research is presented in ways that are most appropriate for the various stakeholders identified during the stakeholder mapping. The data collected in the study along with the study instruments will be made publicly available following the publication of the main study findings, based on approved data sharing agreements.
